# Efficacy of various adjuvant chemotherapy methods in preventing liver metastasis from potentially curative colorectal cancer: A systematic review network meta‐analysis of randomized clinical trials

**DOI:** 10.1002/cam4.5157

**Published:** 2022-08-22

**Authors:** Xianwei Liu, Qisheng Liu, Xiaoyu Wu, Wenbing Yu, Xinmin Bao

**Affiliations:** ^1^ Department of General Surgery Jiujiang First People's Hospital Jiujiang Jiangxi China

**Keywords:** chemotherapy administration, network meta‐analysis, non‐metastatic colorectal cancer, randomized controlled trials

## Abstract

**Purpose:**

Various chemotherapy administration methods have been used to prevent liver metastasis (LM) in patients with colorectal cancer (CRC). This network meta‐analysis evaluated the efficacy of these different methods in preventing LM in CRC patients who underwent curative surgery.

**Method:**

A systematic search of randomized controlled trials reporting the efficacy of various adjuvant chemotherapy methods in patients with colorectal cancer who underwent curative surgery was conducted. The primary outcome was the LM rate.

**Results:**

This network meta‐analysis included 19 studies reporting on 12,588 participants, comparing portal vein infusion chemotherapy (PVIC) versus hepatic arterial infusion chemotherapy (HAIC) versus systematic chemotherapy (SC) versus surgery alone. The HAIC group had the lowest LM rate when compared to the other three groups (odds ratio [OR] of PVIC vs. HAIC: 1.86; OR of SC vs. HAIC: 1.98; and HAIC vs. surgery alone: 0.43). The LM rate did not differ significantly between PVIC, SC, and surgery alone. The recurrence rates were lower for PVIC and HAIC than for surgery alone (the ORs for PVIC and HAIC were 0.73 [95% CI: 0.58–0.92] and 0.45 [95% CI: 0.26–0.77]). The mortality rates of patients undergoing PVIC and HAIC were lower than that of patients undergoing surgery alone (the ORs for PVIC and HAIC were 0.77 [95% CI: 0.64–0.93] and 0.49 [95% CI: 0.24–0.98]). Anastomotic leakage, cardiopulmonary leakage, diarrhea, nausea and vomiting, oral ulceration, wound infection, or ileus did not differ significantly between the four groups. PVIC showed the highest hepatic toxicity rate compared to those for SC, HAIC, and surgery alone.

**Conclusion:**

HAIC might be a satisfactory method for preventing LM in patients with CRC undergoing curative surgery.

## Graphical abstract

We compared four different methods (portal vein infusion chemotherapy, hepatic arterial infusion chemotherapy, systematic chemotherapy, and surgery alone) for preventing liver metastasis in patients with colorectal cancer undergoing curative surgery by means of a network meta‐analysis and concluded that hepatic arterial infusion chemotherapy might be a satisfactory method.

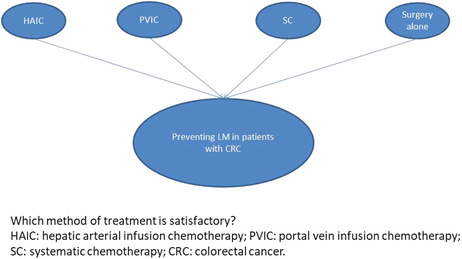

## 1 INTRODUCTION

Distant metastasis is the most common cause of death from colorectal cancer (CRC).^1^ Liver metastasis (LM) is the most common distant metastasis, accounting for approximately 75% of all patients with metastasis, with a five‐year survival rate of only 10%–15%.^2^, ^3^, ^4^, ^5^, ^6^, ^7^ Therefore, preventing LM may be an effective method to improve the prognosis of patients with CRC.

Adjuvant chemotherapy (AC) improves postoperative survival by eliminating micrometastases deposits in some patients with cancer who may relapse.^8^ However, the optimal time for AC in patients with CRC after surgery is unclear. A systematic review of retrospective studies showed that the relative overall survival (OS) rate decreased by 14% every 4 weeks after the delay in AC initiation.^9^ Thus, AC should be administered as soon as possible.

Local chemotherapy is another treatment strategy that can inhibit the proliferation of tumor cells if administered early.^8^ Extrahepatic metastasis often occurs within a short time after LM of CRC.^10^

Therefore, portal vein infusion chemotherapy (PVIC), hepatic arterial infusion chemotherapy (HAIC), and systemic chemotherapy (SC) are the three major methods of AC.^8^, ^11^ Five‐fluorouracil delivered locally rather than systemically provides higher levels of active 5‐fluorouracil metabolites to the liver. However, study results have been inconsistent.^11^ Most studies have only compared the efficacy between two treatment methods or between a certain treatment method and operation alone. No systematic comparison of these four methods (including operation alone) has been performed. Moreover, the effect and safety of regional chemotherapy remain unknown.

Currently, the preferred AC method and its safety remain controversial. Therefore, we performed this network meta‐analysis to evaluate the comparative efficacy of HAIC, PVIC, SC, and no AC for preventing LM in patients with potentially curative CRC.

## 2 METHODS

### 2.1 Search strategy

This systematic review and network meta‐analysis was performed according to the guidelines from the Preferred Reporting Project statement.^12^ A systematic search of randomized clinical trials (RCTs) reporting the efficacy of various adjuvant chemotherapy methods for patients with CRC who underwent curative surgery was conducted in the PubMed, Web of Science, Embase and Cochrane Central Register of Controlled Trials databases. The search strategy is presented in Table S1. The final search was performed on February 10, 2022. A manual search was also performed by based on the reference lists of eligible studies and relevant review articles.

### 2.2 Eligibility criteria

This network meta‐analysis included only RCTs comparing HAIC, PVIC and/or SC alone for preventing LM in patients with non‐metastatic CRC who underwent curative surgery. The following studies were excluded: (1) case reports; (2) reviews, meta‐analyses, conference reports, abstracts or letters; (3) studies focused on the mechanism or functions; and (4) studies without detailed data (Studies which did not provide LM rates or those from which the LM rates could not be extracted.)

### 2.3 Data extraction

Two authors independently reviewed the identified studies and extracted data from the included RCTs. Any disagreements were resolved by a third reviewer. The pre‐specified data elements were extracted from each trial using a structured data abstraction form, including the first author's name, year of publication, country, sample size, interventions, and outcomes of interest.

### 2.4 Outcomes of interest

The primary outcome was the LM rate. The secondary outcomes included recurrence rate, mortality rate, side effects, OS, and disease‐free survival (DFS).

#### 2.4.1 Publication bias and quality assessment

Publication bias was assessed using comparison‐adjusted funnel plot. The methodological qualities of the included RCTs were assessed using the modified Jadad score system.^13^

### 2.5 Statistical analysis

We extracted the hazard ratios (HRs) from the included trials. For articles that did not report HRs, we extracted the HRs from the K‐M curves using Engauge Digitizer version 4.1.^14^ Logarithm‐transformed HRs was pooled using the DerSimonian and Laird random‐effects model.^15^ The HRs was analyzed using OpenBUGS version 3.1.2^16^ (members of OpenBUGS Project Management Group; www.openbugs.net).

A 0.5 zero‐cell correction was applied to all dichotomous variables before performing the meta‐analysis.^17^ The treatment strategies were ranked using surface under cumulative ranking (SUCRA) probabilities and ranking plots.^18^ Higher SUCRA scores and ranking plots corresponded to better efficacy.^18^ A comparison‐adjusted funnel plot was constructed to investigate potential small‐study effects.^19^ The frequentist network meta‐analysis for dichotomous variables, analysis of SUCRA scores, ranking plots, and assessment of publication bias were all performed in STATA (StataCorp, College Station, Texas, USA) using the mvmeta and network commands. Statistical significance was set at *p* < 0.05.^18^

## 3 RESULTS

### 3.1 Study selection

**FIGURE 1:**
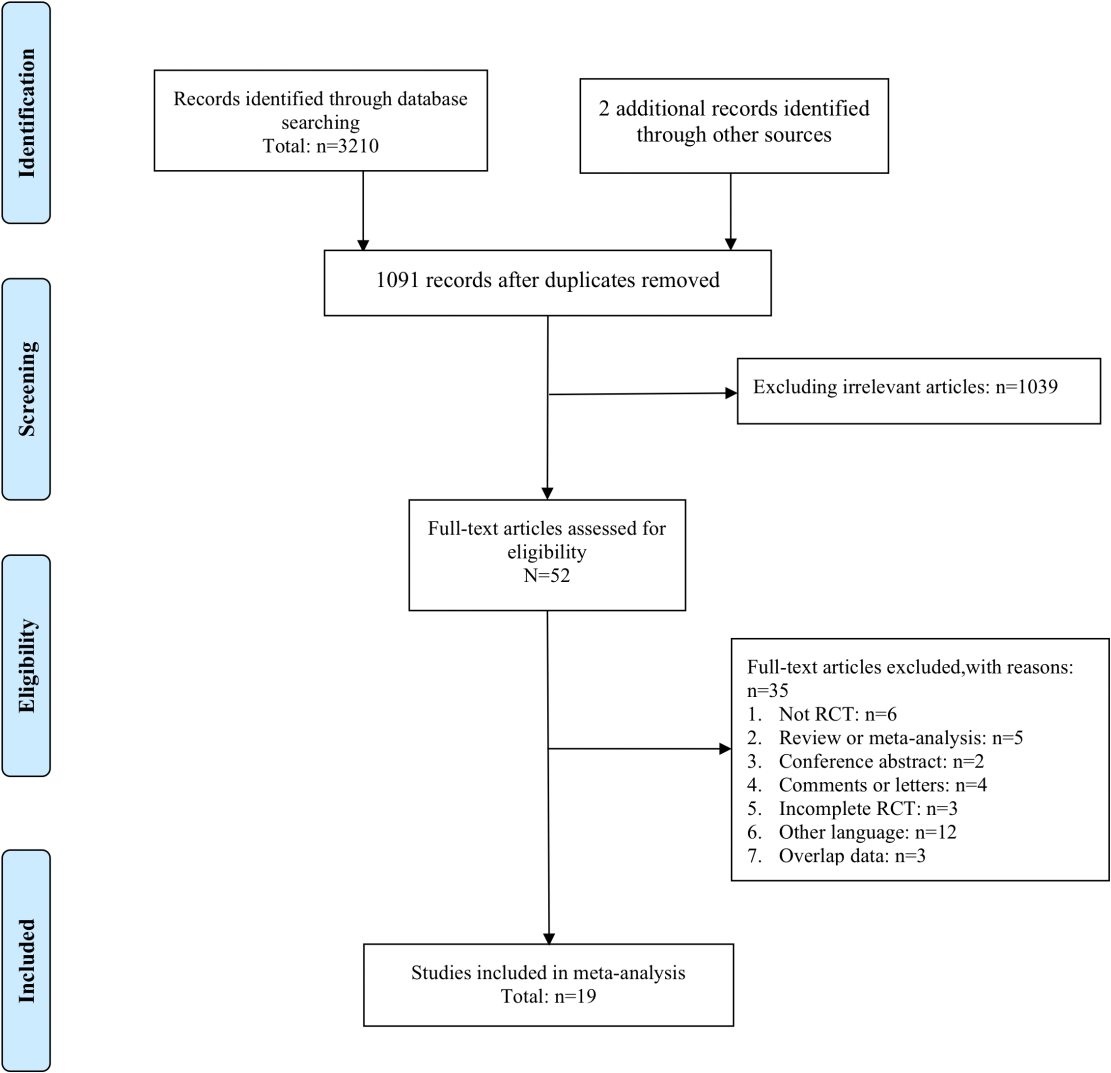
Studies screening according to PRISMA.

A flow diagram of the study selection is shown in Figure 1. A total of 3210 articles were identified, and two additional records were included through a manual search. After removing duplicate reports, 1091 records remained. After reviewing the titles and abstracts, 1039 articles were excluded. After assessing the full texts of the remaining 52 articles, 35 articles were excluded (Figure 1). Only 19 studies met the eligibility criteria and were included in the final analysis.^8^, ^11^, ^20^, ^21^, ^22^, ^23^, ^24^, ^25^, ^26^, ^27^, ^28^, ^29^, ^30^, ^31^, ^32^, ^33^, ^34^, ^35^, ^36^ Gray literature was also searched; however, none met the inclusion criteria.

### 3.2 Study characteristics

**FIGURE 2:**
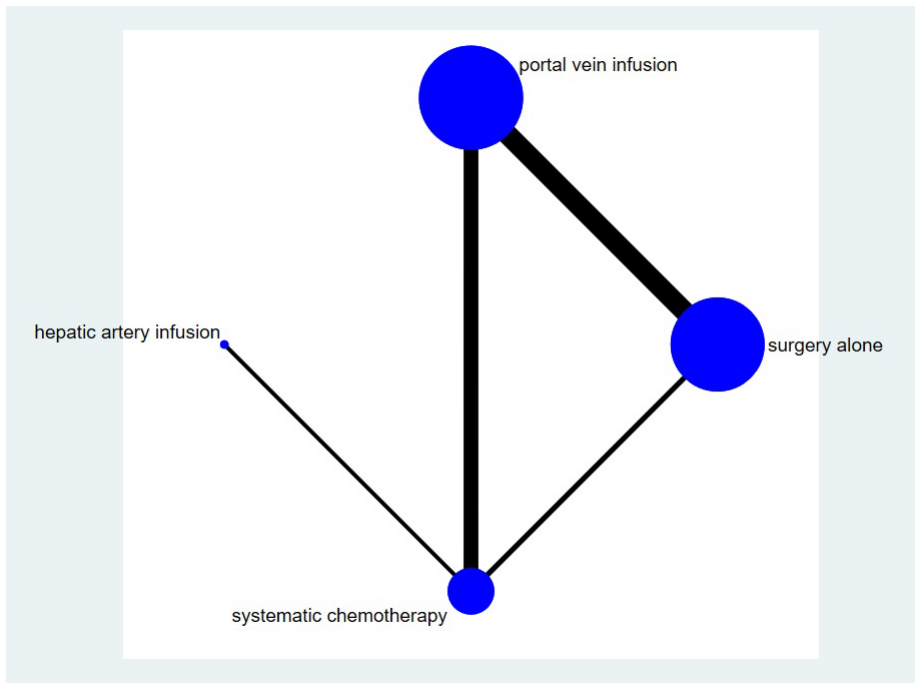
Network plot for eligible comparisons: The size of the nodes is proportional to the number of patients (*n*) randomized to receive the treatment. The width of the lines is proportional to the number of trials (in parentheses) comparing the connected treatment strategies.

The 19 trials comprised 12,588 patients. The ages of the patients in these trials ranged from 18 to 86 years. The characteristics of the included studies are presented in Table 1. Among these, 16 RCTs reported PVIC, three RCTs reported HAIC, seven RCTs reported SC, and 14 RCTs reported surgery alone. The network plots are shown in Figure 2.

**TABLE 1:** Characteristics of all included articles

Study	Year	Country	No. of patients (M/F)	Treatment	Intervention	Timing of intervention	No. of LM	DFS (HR/95% confidence interval)	OS (HR/95% confidence interval)
Taylor et al.	1979	UK	133 (78/55)	PVIC versus Surgery alone	1 g 5‐Fu	7 days post‐operation	PVIC: 2/64; Surgery alone: 13/69	—	—
Taylor et al.	1985	UK	244 (133/111)	PVIC versus Surgery alone	1 g 5‐Fu	7 days post‐operation	PVIC: 5/117; Surgery alone: 22/127	—	0.56 (0.35–0.89)
Metzger et al.	1987	Switzerland	378 (297/179)	PVIC versus Surgery alone	5‐Fu, 500 mg/m^2^	7 days post‐operation	PVIC: 14/191; Surgery alone: 18/187	—	—
Wereldsma et al.	1989	Netherlands	201 (102/99)	PVIC versus Surgery alone	1 g 5‐Fu	7 days post‐operation	PVIC: 4/99; Surgery alone: 18/102	0.29 (0.14–0.61)	0.76 (0.45–1.3)
Beart et al.	1990	US	219 (−/−)	PVIC versus Surgery alone	5‐Fu, 500 mg/m^2^	7 days post‐operation	PVIC:14/110; Surgery alone: 16/109	0.88 (0.50–1.54)	1.0 (0.63–1.60)
Wolmark et al.	1990	US	901 (527/374)	PVIC versus Surgery alone	5‐Fu, 600 mg/m^2^	7 days post‐operation	PVIC: 31/442; Surgery alone: 27/459	1.26 (0.97–1.63)	1.25 (0.90–1.73)
Fielding et al.	1992	UK	275 (−/−)	PVIC versus Surgery alone	1 g 5‐Fu	7 days post‐operation	—	—	0.87 (0.58–1.32)
Yasuo et al.	1994	Japan	1805 (1024/781)	PVIC with SC versus SC versus Surgery alone	PVIC: MMC 12 mg/m^2^ SC: MMC 6 mg/m^2^ with 5‐Fu 200 mg/day.	PVIC:intra‐operation; SC: MMC twice weekly and then three times bimonthly for six months intermittent iv.	PVIC with SC: 79/609; SC: 76/624; Surgery alone: 93/572	—	—
Urban et al.	1995	Switzerland	505 (277/228)	PVIC versus Surgery alone	MMC 10 mg/m^2^ plus 5‐Fu 500 mg/m^2^	MMC one dose with 5‐Fu 7 days post‐operation	PVIC: 44/252; Surgery alone: 54/253	0.79 (0.62–1.00)	0.74 (0.57–0.97)
Nitti et al.	1996	Italy	142 (68/74)	PVIC versus Surgery alone	5‐Fu 500 mg/m^2^	7 days post‐operation	PVIC:8/70; Surgery alone: 11/72	1.0 (0.63–1.59)	1.0 (0.61–1.63)
Rougier et al.	1998	France	1194 (601/593)	PVIC versus Surgery alone	5‐Fu 500 mg/m^2^	7 days post‐operation	PVIC: 64/595; Surgery alone: 63/599	1.02 (0.34–3.05)	1.06 (0.85–1.32)
Focan et al.	2000	Belgium	260 (132/128)	PVIC versus SC	PVIC:5‐Fu 500 mg/m^2^ plus MMC 10 mg/m^2^; SC: 5‐Fu‐based chemotherapy (dose N/A)	PVIC: 5‐Fu continuous infusion for 7 days and MMC at day 7; SC: six courses	PVIC: 18/130; SC: 16/130	1.38 (0.29–6.46)	1.08 (0.23–5.03)
James et al.	2003	UK	3583 (2112/1471)	PVIC versus Surgery alone	1 g 5‐Fu	7 days post‐operation	—	0.90 (0.78–1.04)	0.94 (0.83–1.06)
Sadahiro et al.	2004	Japan	305 (185/120)	HAIC versus Surgery alone	5‐Fu 250 mg/day	7 days pre‐operation and 14 days post‐operation	HAIC: 20/150; Surgery alone: 42/155	0.40 (0.24–0.64)	0.37 (0.21–0.67)
Labianca et al.	2004	Italy	727 (374/353)	PVIC versus SC	PVIC: 5‐Fu 500 mg/m^2^; SC: leucovorin 100 mg/m^2^ daily plus 5‐Fu 370 mg/m^2^ daily on days 1 through 5	PVIC: 7 days post‐operation SC: six 28‐day courses	PVIC: 24/369; SC: 29/358	0.68 (0.63–0.72)	0.74 (0.69–0.79)
Xu et al.	2007	China	222 (125/97)	HAIC with SC versus SC	HAIC: FUDR 500 mg plus oxaliplatin 50 mg; SC: FOLFOX4	HAIC: at the 7 day before operation; SC: standard FOLFOX4	HAIC with SC: 16/110; SC: 23/112	0.61 (0.51–0.79)	0.51 (0.32–0.67)
Laffer et al.	2008	Switzerland	769 (446/323)	PVIC versus SC versus Surgery alone	PVIC: 5‐Fu 500 mg/m^2^ plus MMC 10 mg/m^2^ SC: the same as PVIC	PVIC: 5‐Fu 7 days post‐operation and MMC the first day after operation; SC: the same as PVIC	PVIC: 42/254; SC:34/260; Surgery alone: 36/255	1.18 (0.90–1.54)/0.98 (0.66–1.47)	1.21 (0.91–1.62)/1.03 (0.76–1.38)
Chang et al.	2016	China	237 (138/99)	PVIC with SC versus SC	PVIC: FUDR 1000 mg and oxaliplatin 100 mg SC: mFOLFOX6	PVIC: one dose intra‐operation SC: standard mFOLFOX6	PVIC with SC: 6/118; SC: 12/119	0.66 (0.16–0.90)	0.33 (0.182–1.231)
Zhu et al.	2021	China	688 (389/299)	HAIC with SC versus SC	HAIC: FUDR 650 mg/m^2^ and oxaliplatin 75 mg/m^2^ SC: mFOLFOX6	PVIC: at the 7 day before operation SC: standard mFOLFOX6	HAIC with SC: 24/341; SC: 56/347	0.61 (0.46–0.81)	0.61 (0.43–0.86)

Abbreviations: DFS, disease‐free survival; F, female; FUDR, fluorodeoxyuridine; HAIC, hepatic arterial infusion chemotherapy; HR, hazard ratio; M, male; MMC, mitomycin; NO., Number; OS, overall survival; PVIC, portal vein infusion chemotherapy; SC, systematic chemotherapy; UK, United Kingdom; US, United States.

### 3.3 Quality assessment

The risk of bias for each trial is summarized in Table S2. All 19 studies were considered as high quality (scores of 3–5).

### 3.4 LM rates

Of the 19 studies, 17 reported LM rates. The network meta‐analysis of LM rates is shown in Table 2. Of the four treatment groups, HAIC was superior to the other three (odds ratio [OR] of PVIC vs. HAIC: 1.86; OR of SC vs. HAIC: 1.98; and HAIC vs. surgery alone: 0.43). No significant differences were observed among PVIC, SC, and surgery alone. HAIC, PVIC, SC, and surgery alone ranked from best to worst in preventing LM according to SUCRA (Figure S1).

**TABLE 2:** ORs of the rate of liver metastasis

PVIC			
0.94 (0.69–1.27)	SC		
1.86 (1.15–3.00)	1.98 (1.28–3.07)	HAIC	
0.79 (0.62–1.01)	0.84 (0.63–1.13)	0.43 (0.28–0.65)	Surgery alone

Abbreviations: HAIC, hepatic arterial infusion chemotherapy; PVIC, portal vein infusion chemotherapy; SC, systematic chemotherapy.

Among the 17 studies, seven focused on colon cancer. Pairwise comparisons showed that HAIC, PVIC, SC, and surgery alone ranked from best to worst in the subgroup of colon cancer (Figure S2). A subgroup analysis of the rectum could not be performed owing to the limited number of studies.

### 3.5 Recurrence rates

Thirteen studies involving 8029 patients reported recurrence rates. Compared to surgery alone, the ORs for PVIC, HAIC and SC were 0.73 (95% CI: 0.58–0.92), 0.45 (95% CI: 0.26–0.77) and 0.74 (95% CI: 0.54–1.02), respectively. No significant difference was observed between PVIC, SC, and HAIC. Pairwise comparisons of these four treatments are presented in Table 3. SUCRA and rank plotting analysis revealed that HAIC, PVIC, SC and surgery alone ranked from the best to worst in preventing recurrence (Figure S3).

**TABLE 3:** ORs of recurrence rate

PVIC			
0.98 (0.73–1.31)	SC		
1.62 (0.93–2.82)	1.66 (0.99–2.78)	HAIC	
0.73 (0.58–0.92)	0.74 (0.54–1.02)	0.45 (0.26–0.77)	Surgery alone

Abbreviations: HAIC, hepatic arterial infusion chemotherapy; PVIC, portal vein infusion chemotherapy; SC, systematic chemotherapy.

### 3.6 Patients' mortality rates

Seventeen studies containing 12,261 patients presented the patient mortality rates. Compared with surgery alone, the ORs for PVIC, HAIC and SC were 0.77 (95% CI: 0.64–0.93), 0.49 (95% CI: 0.24–0.98) and 0.80 (95% CI: 0.59–1.08), respectively. No significant differences were observed among the PVIC, SC, and HAIC groups. The pairwise comparisons of the four treatments are presented in Table 4. According to SUCRA and rank plotting analysis, HAIC, PVIC, SC and surgery alone ranked from best to worst in terms of decreasing patient mortality (Figure S4).

**TABLE 4:** ORs of patients mortality rate

PVIC			
0.96 (0.73–1.28)	SC		
1.58 (0.79–3.13)	1.63 (0.87–3.06)	HAIC	
0.77 (0.64–0.93)	0.80 (0.59–1.08)	0.49 (0.24–0.98)	Surgery alone

Abbreviations: HAIC, hepatic arterial infusion chemotherapy; PVIC, portal vein infusion chemotherapy; SC, systematic chemotherapy.

### 3.7 Side effects

Four studies reported hepatic toxicity. The network meta‐analysis of LM rates is shown in Table 5. Among these four treatment groups, PVIC showed a significantly higher hepatic toxicity rate than those for SC (OR = 3.23), HAIC (OR = 3.80), and surgery alone (OR = 13.47). The hepatic toxicity rates were similar between the SC and HAIC groups; however, both groups had higher hepatic toxicity rates than that for surgery alone (OR of SC vs. surgery alone: 4. 17, and OR of HAIC vs. surgery alone: 3.54). In addition, anastomotic leakage, cardiopulmonary leakage, diarrhea, nausea and vomiting, oral ulceration, wound infection, and ileus did not differ between the four groups.

**TABLE 5:** ORs of hepatic toxicity

PVIC			
3.23 (1.03–10.13)	SC		
3.80 (1.01–14.34)	1.18 (0.60–2.31)	HAIC	
13.47 (2.84–63.82)	4.17 (91.45–11.98)	3.54 (1.57–7.97)	Surgery alone

Abbreviations: HAIC, hepatic arterial infusion chemotherapy; PVIC, portal vein infusion chemotherapy; SC, systematic chemotherapy.

### 3.8 Publication bias

A comparison‐adjusted funnel plot showed no publication bias (Figure S5).

### 3.9 Os and DFS

Sixteen articles including 10,472 patients reported OS. Compared to surgery alone, the HRs for PVIC, HAIC and SC were 0.95 (95% CI: 0.87–1.03), 0.76 (95% CI: 064–0.89), and 1.03 (95% CI: 0.88–1.18), respectively.

Fourteen studies including 9953 patients reported DFS. Compared to surgery alone, the HRs for PVIC, HAIC and SC were 0.93 (95% CI: 0.82–1.05), 0.78 (95% CI: 0.63–0.93) and 1.01 (0.81–1.20), respectively.

## 4 DISCUSSION

Many patients experience relapse after radical surgery and standard SC.^37^, ^38^ LM is the most common form of cancer recurrence.^38^ Therefore, preventing LM or extending the time to LM is a clinical research hotspot in resectable CRC.^11^ PVIC, HAIC and SC are common clinical treatments for LM prevention.^39^ However, the clinical efficacy and side effects of these methods remain controversial. Therefore, we performed this network meta‐analysis, which, to our knowledge, is the first network meta‐analysis in this field.

The results of our meta‐analysis indicated that HAIC had the best effect in terms of the cumulative rate of LM, death, recurrence and complications during the follow‐up period. This was also true in the subgroup analysis of the cumulative rate of LM in colon cancer. PVIC showed advantages in terms of mortality and recurrence; however, PVIC showed unsatisfactory performance in terms of the HR of OS and DFS. Regarding side effects, PVIC showed the worst hepatic toxicity, followed by HAIC, SC, and surgery alone.

Considering the special blood supply to the liver, PVIC was first used to prevent LM in patients with CRC.^20^ However, the efficacy of intraoperative or postoperative PVIC in preventing postoperative LM in patients with CRC is controversial; therefore, it is currently rarely performed.^11^ The results of our meta‐analysis suggested that PVIC had no obvious advantages in preventing LM and failed to show satisfactory results in postoperative OS and DFS and even increased the risk of hepatic toxicity. However, PVIC improved the rates of patient mortality and CRC recurrence during the follow‐up period.

HAIC has shown good effects in the treatment of colorectal liver metastasis (CRLM) due to its advantages of targeting the liver and tumors.^11^, ^40^, ^41^ Many patients with unresectable LMs can convert to resectable tumors after HAIC combined with SC, thereby prolonging OS.^42^, ^43^ For patients with potentially curative CRC, the main cause of LM after surgery is liver micrometastases at the time of surgery.^44^ HAIC is based on the liver as a target for treatment, which can effectively prevent and even eliminate liver micrometastases.^11^ This may explain the better preventative effect of HAIC on postoperative LM in patients with CRC. Studies have confirmed the excellent performance of HAIC in reducing postoperative LM, enhancing OS, and improving DFS for patients with resectable CRC in comparisons of surgery plus perioperative HAIC to surgery alone or HAIC plus SC to SC alone.^11^, ^33^, ^35^, ^45^, ^46^ The results of our meta‐analysis also confirmed the significant therapeutic advantage of HAIC in patients with potentially curable CRC without increasing the corresponding side effects.

Despite the potential survival advantages, previous experience has indicated that regional chemotherapy could cause liver‐specific toxicity, increase perioperative morbidity, and preclude liver resection. However, in our study, PVIC, HAIC, and SC showed no significantly worse outcomes in anastomotic leakage, cardiopulmonary, diarrhea, nausea and vomiting, oral ulceration, and wound infection compared to surgery alone. In terms of hepatic toxicity, PIVC showed the worst side effects. HAIC did not show significantly worse hepatic toxicity compared to SC. The main blood supply and nutrients of liver cells originate from the portal vein system, which could explain this phenomenon.

Our study had several strengths. First, to our knowledge, this was the first network meta‐analysis to systematically study all adjuvant chemotherapy methods for preventing LM and to include all published articles on this topic through 2022. Second, we analyzed the efficacy of different treatment methods from multiple dimensions; namely, cumulative rates of LM, HR for OS, HR for DFS, as well as the rates of patient death and CRC recurrence during the follow‐up period and various side effects. Third, the results were consistent across all aspects. HAIC had the lowest hepatic toxicity and best effects in preventing LM in terms of HR for OS, HR for DFS, rate of patient death, and CRC recurrence during the follow‐up period.

However, this study has some limitations. First, the subgroup analyses were insufficient owing to the limited literature and various research methods. For example, it was not possible to perform subgroup analysis according to different drugs and doses. However, several studies have reported that oxaliplatin‐based chemotherapy is more effective and safe than fluorouracil.^47^, ^48^, ^49^, ^50^ We also failed to analyze efficacy in the rectal subgroup, left and right colon subgroups. Patients with rectal or colon cancer showed different incidences of LM, OS, and DFS, which could also be represent in patients with right or left colon cancer.^51^, ^52^ It was also possible to analyze the curative effect according to the tumor stage. Studies have reported significant differences in the curative effect between patients with stage II and III colon cancer.^11^, ^25^, ^27^ Second, the definition of side effects was not uniform across trials. Although we attempted to standardize this definition during data extraction, it may not fully capture all relevant side effects. Third, the time span of the included trials was relatively large, and follow‐up times varied greatly, which have led to a bias in the results of our analyses.

## 5 CONCLUSION

This relatively comprehensive study was the first network meta‐analysis to investigate all adjuvant chemotherapy methods for preventing LM. Our results showed the satisfactory efficiency of HAIC in preventing LM in patients with potentially radically curable CRC compared to other treatment methods. These findings may help guide treatment selection in clinical practice.

## FUNDING INFORMATION

Not applicable.

## CONFLICT OF INTEREST

The authors declare that they have no competing interests.

## ETHICS APPROVAL

Not applicable.

## CONSENT FOR PUBLICATION

Not applicable.

## Supporting information


Figure S1

Figure S2

Figure S3

Figure S4

Figure S5

Table S1

Table S2

## Data Availability

The datasets used and/or analyzed during the current study are available from the corresponding author on reasonable request.
