# Supplementary material for: Efficacy of various adjuvant chemotherapy methods in preventing liver metastasis from potentially curative colorectal cancer: A systematic review network meta‐analysis of randomized clinical trials
Source: Cancer Med. 2022 Aug 22;12(3):2238–47. doi: 10.1002/cam4.5157 (PMC9939089; doi:10.1002/cam4.5157)
Supplement: Supplementary file 1 — Figure S1 [file CAM4-12-2238-s004.docx]

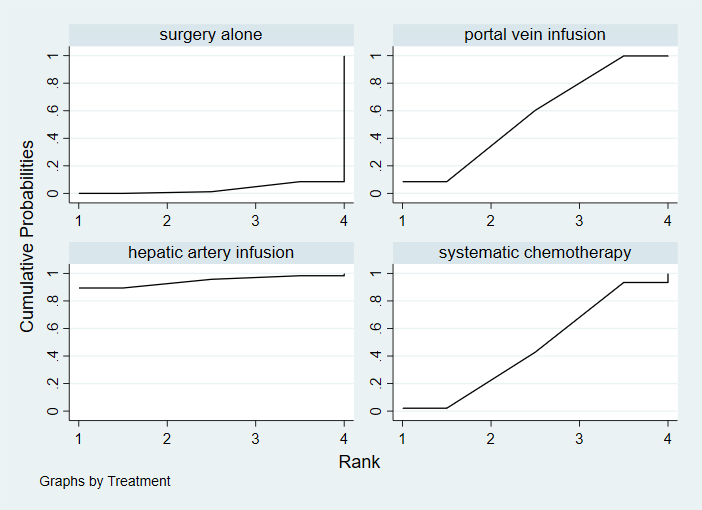


**Figure S1:** SUCRA (surface under the cumulative ranking curve) for the efficiency of decreasing liver metastasis during follow-up period by different treatment methods.

Ranking indicates the probability of being the best (hepatic artery infusion), second best (portal vein infusion), third best (systemic chemotherapy) and least good (surgery alone).
