# Supplementary material for: Efficacy of various adjuvant chemotherapy methods in preventing liver metastasis from potentially curative colorectal cancer: A systematic review network meta‐analysis of randomized clinical trials
Source: Cancer Med. 2022 Aug 22;12(3):2238–47. doi: 10.1002/cam4.5157 (PMC9939089; doi:10.1002/cam4.5157)
Supplement: Supplementary file 2 — Figure S2 [file CAM4-12-2238-s001.docx]

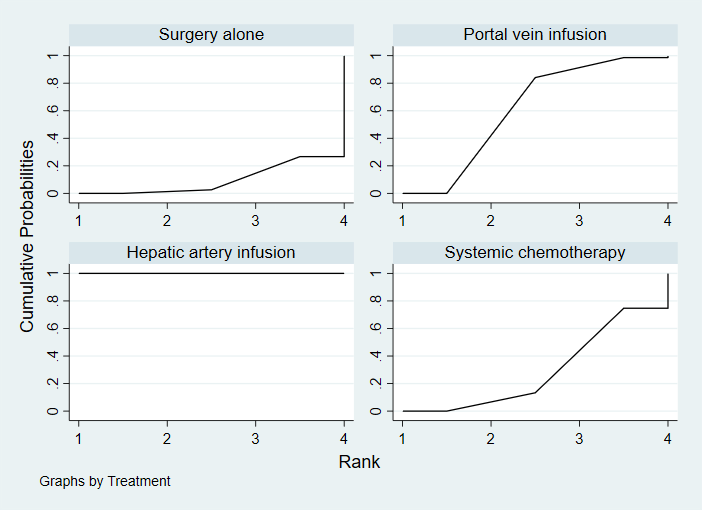


**Figure S2:** SUCRA (surface under the cumulative ranking curve) for the efficiency of decreasing liver metastasis during follow-up period by different treatment methods in the subgroup of colon cancer.

Ranking indicates the probability of being the best (hepatic artery infusion), second best (portal vein infusion), third best (systemic chemotherapy) and least good (surgery alone).
