# Supplementary figures and images for: Efficacy of various adjuvant chemotherapy methods in preventing liver metastasis from potentially curative colorectal cancer: A systematic review network meta‐analysis of randomized clinical trials
Source: Cancer Med. 2022 Aug 22;12(3):2238–47. doi: 10.1002/cam4.5157 (PMC9939089; doi:10.1002/cam4.5157)

**
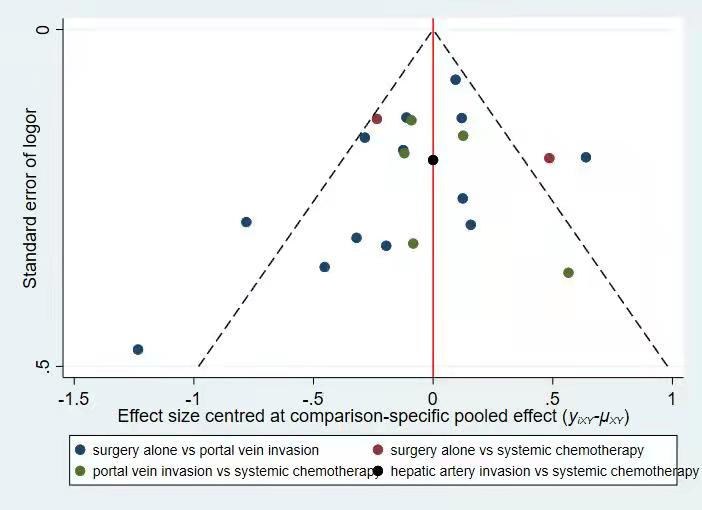
**

**Figure S5:** Comparison-adjusted funnel plot.

Supplement: Supplementary file 5 — Figure S5 [file CAM4-12-2238-s006.docx]
