# Supplementary material for: Efficacy of various adjuvant chemotherapy methods in preventing liver metastasis from potentially curative colorectal cancer: A systematic review network meta‐analysis of randomized clinical trials
Source: Cancer Med. 2022 Aug 22;12(3):2238–47. doi: 10.1002/cam4.5157 (PMC9939089; doi:10.1002/cam4.5157)
Supplement: Supplementary file 6 — Table S1 [file CAM4-12-2238-s003.docx]

Table S1. Search Strategy.

| Database | Search Strategy | Number |
| --- | --- | --- |
| Pubmed | ((((((((((((((colon cancer) OR colon cancers) OR rectal cancer) OR rectal cancers) OR colorectal cancer) OR colorectal cancers) OR colon carcinoma) OR colon carcinomas) OR rectal carcinoma) OR rectal carcinomas) OR colorectal carcinoma) OR colorectal carcinomas) AND ((((((((randomized controlled trial) OR randomized controlled trials) OR clinical trial) OR clinical trials) OR RCT) OR RCTs) OR randomized trial) OR randomized trials)) AND ((chemotherapy) OR (adjuvant therapy))) AND (((((((((Intraportal) OR (portal vein)) OR (portal veins)) OR (hepatic arterial)) OR (hepatic artery)) OR (hepatic arteries)) OR (HAI)) OR (PVI)) OR (portal-vein)) | 689 |
| Web of Science | (((((((((((ALL=(colon cancer)) OR ALL=(colon cancers)) OR ALL=(rectal cancer)) OR ALL=(rectal cancers)) OR ALL=(colorectal cancer)) OR ALL=(colorectal cancers)) OR ALL=(colon carcinoma)) OR ALL=(colon carcinomas)) OR ALL=(rectal carcinoma)) OR ALL=(rectal carcinomas)) OR ALL=(colorectal carcinoma)) OR ALL=(colorectal carcinomas) AND (((((((ALL=(randomized controlled trial)) OR ALL=(randomized controlled trials)) OR ALL=(clinical trial)) OR ALL=(clinical trials)) OR ALL=(RCT)) OR ALL=(RCTs)) OR ALL=(randomized trial)) OR ALL=(randomized trials) AND (ALL=(chemotherapy)) OR ALL=(adjuvant therapy) AND ((((((((ALL=(Intraportal)) OR ALL=(portal vein)) OR ALL=(portal veins)) OR ALL=(hepatic arterial)) OR ALL=(hepatic artery)) OR ALL=(hepatic arteries)) OR ALL=(HAI)) OR ALL=(PVI)) OR ALL=(portal-vein) | 915 |
| Ovid | (colon cancer or colon cancers or rectal cancer or rectal cancers or colorectal cancer or colorectal cancers or colon carcinoma or colon carcinomas or rectal carcinoma or rectal carcinomas or colorectal carcinoma or colorectal carcinomas).af. AND (randomized controlled trial or randomized controlled trials or clinical trial or clinical trials or RCT or RCTs or randomized trial or randomized trials).af. AND (chemotherapy or adjuvant therapy).af. AND (Intraportal or portal vein or portal veins or hepatic arterial or hepatic artery or hepatic arteries or HAI or PVI or portal-vein).af. | 1243 |
| Cochrance | ((((((((((((((colon cancer) OR colon cancers) OR rectal cancer) OR rectal cancers) OR colorectal cancer) OR colorectal cancers) OR colon carcinoma) OR colon carcinomas) OR rectal carcinoma) OR rectal carcinomas) OR colorectal carcinoma) OR colorectal carcinomas) AND ((((((((randomized controlled trial) OR randomized controlled trials) OR clinical trial) OR clinical trials) OR RCT) OR RCTs) OR randomized trial) OR randomized trials)) AND ((chemotherapy) OR (adjuvant therapy))) AND (((((((((Intraportal) OR (portal vein)) OR (portal veins)) OR (hepatic arterial)) OR (hepatic artery)) OR (hepatic arteries)) OR (HAI)) OR (PVI)) OR (portal-vein)) | 363 |
