# Supplementary material for: Efficacy of various adjuvant chemotherapy methods in preventing liver metastasis from potentially curative colorectal cancer: A systematic review network meta‐analysis of randomized clinical trials
Source: Cancer Med. 2022 Aug 22;12(3):2238–47. doi: 10.1002/cam4.5157 (PMC9939089; doi:10.1002/cam4.5157)
Supplement: Supplementary file 7 — Table S2 [file CAM4-12-2238-s007.docx]

Table S2 Evaluation of methodological qualities of included randomized controlled trials.

| Items/author | Taylor1 | Taylor2 | Metzger | Wereldsma | Beart | Wolmark | Fielding | Yasuo | Urban | Nitti |
| --- | --- | --- | --- | --- | --- | --- | --- | --- | --- | --- |
| Described as randomized | 1 | 1 | 1 | 1 | 1 | 1 | 1 | 1 | 1 | 1 |
| Appropriate randomization method described | 1 | 1 | 1 | 1 | 1 | 1 | 1 | 1 | 1 | 1 |
| Subject blinded to intervention | 1 | 1 | 1 | 0 | 1 | 1 | 1 | 1 | 1 | 1 |
| Evaluator blinded to intervention | 1 | 0 | 1 | 1 | 1 | 0 | 1 | 1 | 0 | 0 |
| Description of withdrawals and dropouts | 0 | 1 | 1 | 1 | 1 | 1 | 1 | 0 | 1 | 1 |
| Total Score | 4 | 4 | 5 | 4 | 5 | 3 | 5 | 4 | 4 | 4 |

| Items/author | Rougier | Focan | James | Sadahiro | Labianca | Xu | Laffer | Chang | Zhu |
| --- | --- | --- | --- | --- | --- | --- | --- | --- | --- |
| Described as randomized | 1 | 1 | 1 | 1 | 1 | 1 | 1 | 1 | 1 |
| Appropriate randomization method described | 1 | 1 | 1 | 1 | 1 | 1 | 1 | 1 | 1 |
| Subject blinded to intervention | 0 | 1 | 1 | 1 | 1 | 1 | 1 | 1 | 1 |
| Evaluator blinded to intervention | 1 | 1 | 1 | 1 | 1 | 1 | 1 | 1 | 1 |
| Description of withdrawals and dropouts | 1 | 1 | 1 | 0 | 1 | 1 | 1 | 0 | 1 |
| Total Score | 4 | 5 | 5 | 4 | 5 | 5 | 5 | 4 | 5 |

Taylor1 was published in 1979, Taylor2 was published in1985. Methodological qualities of included randomized controlled trials were assessed by modified Jadad score system. Total score, 5; 1-2, low quality trial; 3-5, high quality trial.
